# Pathophysiology of hypercalciuria in children

**DOI:** 10.1007/s00467-007-0482-6

**Published:** 2007-10-01

**Authors:** Tarak Srivastava, Uri S. Alon

**Affiliations:** Section of Nephrology, Bone and Mineral Disorder Clinic, The Children’s Mercy Hospital and Clinics, University of Missouri, 2401 Gillham Road, Kansas City, MO 64108 USA

**Keywords:** Hypercalciuria, Children

## Abstract

Urinary excretion of calcium is the result of a complex interplay between three organs—namely, the gastrointestinal tract, bone, and kidney—which is finely orchestrated by multiple hormones. Hypercalciuria is believed to be a polygenic trait and is influenced significantly by diet. This paper briefly reviews calcium handling by the renal tubule in normal and in hereditary disorders as it relates to the pathophysiology of hypercalciuria. The effects of dietary sodium, potassium, protein, calcium, and phosphate on calcium excretion, and the association of hypercalciuria with bone homeostasis is discussed, leading to recommendations on means to address excessive urinary calcium excretion.

## Introduction

Albright et al. [1] first introduced the term idiopathic hypercalciuria to describe patients with recurrent urolithiasis who had elevated urinary calcium excretion without concomitant hypercalcemia. The etiology of hypercalciuria is complex given that urinary excretion of calcium is the end result of an interplay between three organs—namely, the gastrointestinal tract, bone, and kidney—which is further orchestrated by hormones, such as 1,25-dihydroxyvitamin D_3_ (1,25-(OH)_2_D_3_), parathyroid hormone (PTH), calcitonin, fibroblast growth factor (FGF-23), etc. Often, a primary defect in one organ induces compensatory mechanisms in the remaining two organs, such as increased absorption of calcium in the gut secondary to a primary renal loss. Hypercalciuria can be either idiopathic or secondary. In this review, idiopathic hypercalciuria and the recent developments in hereditary renal tubular disorders associated with hypercalciuria is discussed, providing an insight into the pathophysiology of hypercalciuria. The role of diet in hypercalciuria and the clinical relationship of hypercalciuria with urolithiasis and bone mineral density (BMD) is also discussed.

## Genetics of hypercalciuria

Hypercalciuria is the most common metabolic abnormality detected in children with stones, causing mostly the formation of calcium oxalate stones and to a lesser extent calcium phosphate stones or a mixture of the two [2–4]. The reported incidence for urolithiasis in Icelandic children is 6.3/100,000 children <16 years of age [2]. In adults, Curhan et al. [5] reported 14–27% hypercalciuria in a cohort of control population identified from the three large studies: Nurses’ Health Study I (NHS I), NHS II, and Health Professional Follow-up Study, whereas it was 25–38% in stone formers in the same cohort. Coe et al. [6] assessed that 5% of American women and 12% of men will develop a kidney stone at some time in their life. A positive family history appears to be the single most important risk factor after controlling for known dietary factors [7]. In children with hypercalciuria, the prevalence of urolithiasis in the family is 69% [8]. Reed et al. [9] mapped a defect in three families with severe absorptive hypercalciuria to 1q23.3-q24, and they subsequently sequenced a putative gene (homologous to rat soluble adenylate cyclase gene). They identified 18 base substitutions in the putative gene, four of which increased the relative risk of absorptive hypercalciuria by 2.2- to 3.5-fold [10]. Vezzoli et al. [11] found single nucleotide polymorphism Arg990Gly in the calcium-sensing receptor (CASR) gene to account for 4.1% of total variance in calcium excretion and 12.6% of total variance in calcium excretion if independent variables of sodium excretion, body weight, serum creatinine, and enteral absorption of strontium were added to the multiple regression model. Imamura et al. [12] and Giuffre et al. [13] described three unrelated children with hypercalciuria who have 4q33-qter and 4q31.3-qter deletion, respectively, which raises the potential for a putative gene for hypercalciuria in that region. At this point, hypercalciuric trait is suspected to be polygenic and requires the interaction of genetic and/or environmental factors [14, 15]. A familial clustering of idiopathic calcium nephrolithiasis is frequently observed, most often compatible with an autosomal dominant transmission, but the quantitative genetics of urine calcium excretion has not been established. Loredo-Osti et al. [16] believe that either a mixed codominant/ polygenic model or a single-gene codominant model best determines the estimated inheritable attribute for idiopathic hypercalciuria, and thus it should be feasible to genetically map the quantitative trait locus for idiopathic hypercalciuria.

## Physiology of calcium absorption and its implication in diseases

Calcium exists in three distinct pools in the body, where it is tightly regulated. The largest pool is that in the skeleton in the molar range, followed by the extracellular calcium pool in the millimolar range, and the third is in the intracellular space, which contains no more than 1 μm of calcium in an adult.

### Calcium absorption in the gastrointestinal tract

Calcium absorption in the gastrointestinal tract is a sum of two transport processes: a saturable transcellular absorption that is physiologically regulated by vitamin D, and a nonsaturable paracellular absorption that is dependent on the calcium concentration within the lumen, which in turn depends on dietary calcium load. The nonsaturable paracellular pathway is thought to predominate when the diet is replete in calcium, whereas the saturable vitamin-D-dependent transcellular pathway becomes critical when the dietary calcium is limited.

The active transcellular absorption is mediated by epithelial transient receptor potential (TRP) calcium channels TRPV5 (epithelial calcium channel: ECaCl) and TRPV6 (CaT1 or ECaC2), which are under the regulation of 1,25-(OH)_2_D_3_ [17, 18]. TRPV5 and TRPV6 calcium channels are transmembrane proteins that belong to the superfamily of TRP channels. TRPV5 and TRPV6 have been mapped to chromosomes 7q35 and 7q33-34, respectively, and are believed to be products of evolutionary local gene duplication [19, 20]. TRPV5 and TRPV6 are coexpressed in the intestine and kidney; however, TRPV6 is more abundant and is believed to be the major calcium channel in the intestine [21–23]. TRPV6 is expressed from the esophagus down to the jejunum, whereas TRPV5 is restricted to the duodenum and jejunum. In contrast, TRPV5 is abundantly expressed in the renal distal convoluted duct and connecting tubule, whereas limited expression of TRPV6 is observed in the distal convoluted tubule (DCT), connecting tubule, and collecting duct [22, 23]. In the intestine, TRPV6 is present in a thin layer along the apical membrane of the duodenal villus tip and colocalizes with calbindin-D(9K) and plasma membrane Ca(2+)-ATPase (PMCA), all involved in calcium transport.

### Calcium absorption in the kidney

**Fig. 1:**
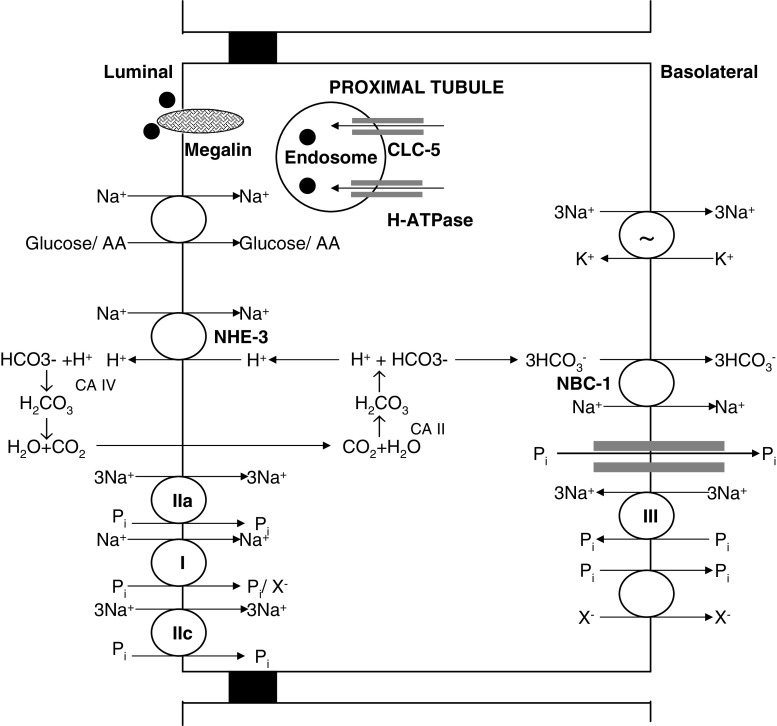
Schematic representation of molecular (or transport) mechanisms in the proximal tubule related to Dent’s disease and hereditary hypophosphatemic rickets with hypercalciuria. • low molecular weight proteins; *NHE-3* Na^+^-H^+^ exchanger; *I, IIa, IIc, and III* Na/Pi type I, type IIa, type IIc and type III cotransporters; *NBC-1* Na^+^-HCO^−^_3_ ${\text{Na}}^{ + }  - {\text{CO}}^{ - }_{3}$ cotransporter; *CLC-5* chloride channel-5; *AA* amino acids; *X* ^−^ anion; *Pi* phosphate; *CA II* cytoplasmic carbonic anhydrase; *CA IV* membrane-bound carbonic anhydrase

**Fig. 2:**
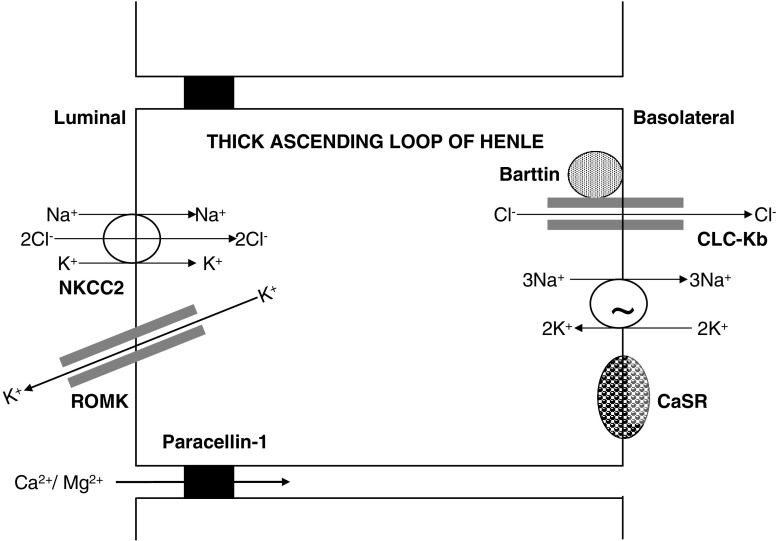
Schematic representation of molecular (or transport) mechanisms in the thick ascending loop of Henle related to Bartter syndrome and familial hypomagnesemia with hypercalciuria and nephrocalcinosis.* NKCC2* Na^+^ - K^+^-2C1^−^ cotransporter, *ROMK* renal outer-medullary potassium channel, *CLC-Kb* chloride channel Kb, *CaSR* calcium-sensing receptor

Only filterable calcium, i.e., non-albumin-bound calcium, is filtered in the glomerulus to the urinary space. The calcium in the glomerular ultrafiltrate is then handled throughout the renal tubule to maintain calcium homeostasis. The retrieval of ∼70% calcium occurs in the proximal tubule and ∼20% in the thick ascending loop of Henle (TALH), predominantly by a paracellular mechanism. The calcium absorption in the proximal tubule occurs mainly from the solvent drag that occurs from salt and water absorption, whereas in the TALH, the paracellular calcium absorption is driven by the lumen-positive potential generated by the sodium absorption from the Na^+^ - K^+^-2C1^−^ cotransporter (NKCC2), renal outer-medullary potassium channel (ROMK), and chloride channel (Figs. 1 and 2) [24]. Thus, calcium absorption in the renal tubule is at the mercy of sodium absorption, which is crucially important in the dietary management of hypercalciuria.

**Fig. 3:**
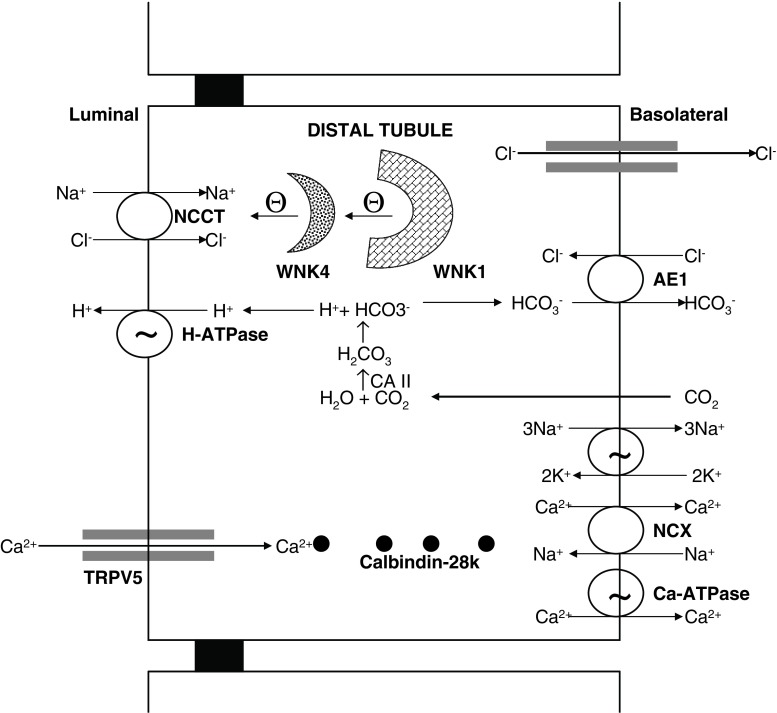
Schematic representation of molecular (or transport) mechanisms in the distal tubule related to pseudohypoaldosteronism (PHA) type II and distal renal tubular acidosis.* NCCT* Na^+^-Cl^−^ cotransporter, *TRPV5* calcium channel, *AE1* Cl^−^-HCO^−^_3_ ${\text{Cl}}^{ - } {\text{HCO}}^{ - }_{3}$ exchanger, *CA II* cytoplasmic carbonic anhydrase, *NCX* Na^+^-Ca^2+^ exchanger, *WNK1* with-no-lysine kinase 1, *WNK4* with-no-lysine kinase 4

The fine tuning of the remaining ∼10% calcium occurs in the DCT, connecting tubule, and initial portion of the cortical collecting duct through an active transcellular pathway (Fig. 3) [25–27]. The molecular nature of the apical epithelial calcium channel TRPV5 was first elucidated by expression cloning and using primary cultures of rabbit connecting tubule [17]. TRPV5 has been identified immunohistochemically in DCT and connecting tubule [22, 23]. TRPV5 colocalizes with calbindin-D(28K), Na(+)-Ca(2+) exchanger (NCX), and PMCA. Calbindin- D(28K) acts as the principal calcium shuttle from the apical to the basolateral surface [22, 23, 26]. The subsequent basolateral calcium transport occurs via both the plasma membrane NCX and PMCA, which are estimated to transport 70% and 30% of calcium, respectively [28, 29]. The calcium transport in the distal tubule is regulated by PTH and 1,25-(OH)_2_D_3_ [28, 29]. Hoenderop et al. [30] demonstrated that mice lacking TRPV5 displayed diminished active calcium absorption despite enhanced vitamin D levels, causing severe hypercalciuria and significant disturbances in bone structure, including reduced trabecular and cortical bone thickness.

#### Proximal tubule in hypercalciuria

The majority (∼70%) of calcium absorption occurs in the proximal tubule in an isosmotic process mainly from the solvent drag that occurs from salt and water absorption [26, 27]. The proximal tubule is critical for bulk absorption of sodium, chloride, phosphate, amino acids, glucose, etc., where the calcium absorption is taking place in an energetically passive mode, and thus a dysfunction as in Fanconi syndrome will result in variable hypercalciuria. Hypercalciuria has been observed in disorders affecting the proximal tubule, such as Lowe syndrome, Wilson’s disease, Tyrosinemia type 1, and glycogen storage disease type 1a. Dent’s disease and hereditary hypophosphatemic rickets with hypercalciuria where new genetic information has become available will be discussed in more detail.

##### Dent’s disease

Dent’s disease is now an accepted name for a group of hereditary tubular disorders, including X-linked recessive nephrolithiasis with renal failure, X-linked recessive hypophosphatemic rickets, and idiopathic low-molecular-weight proteinuria associated with hypercalciuric nephrocalcinosis resulting from mutations in the *CLCN5* gene located on Xp11.22 encoding for the chloride channel 5 (CLC-5) [31, 32]. CLC-5 belongs to a family of nine known voltage-gated, transmembrane-spanning, chloride channel genes. It is localized to endosomes and is expressed in the proximal tubule, TALH, and collecting duct [33, 34]. CLC-5 colocalizes with H^+^-ATPase, and this pairing is important for acidification of endocytotic vesicles [33]. The degradation activity within the endosome in the proximal tubule is critically dependent on the acidification of the endosomal lumen, which is believed to be mediated by CLC-5. The exact mechanism that underlies hypercalciuria in Dent’s disease is still under investigation. CLC-5 defect leads to abnormal regulation of PTH and vitamin D metabolites from diminished recycling of low-molecular-weight proteins, such as luminal PTH receptors, vitamin-D-binding protein, etc.[35–37]. Recent work by Silva et al. [38] demonstrated that the hypercalciuria in the CLC-5 knockout mice on low- and high-calcium diets is of bone and renal origin and is not caused by increased intestinal calcium absorption, despite an elevated serum 1,25-(OH)_2_D_3_.

The phenotypic expression of Dent’s disease is quite wide. The disease is more severe in males and is characterized by hypercalciuria, medullary nephrocalcinosis, nephrolithiasis, low-molecular-weight proteinuria and other tubular dysfunctions, and progressive renal failure. The proximal tubular dysfunction can manifest as glucosuria, aminoaciduria, and phosphaturia [39]. Renal failure occurs in about two thirds of patients with Dent’s disease; the renal function generally begins to decline in the adolescent years and reaches end stage by the fourth decade of life. Serum calcium levels are normal or high-normal, PTH is low, and levels of 1,25-(OH)_2_D_3_ are often elevated [31, 39–41]. Hypercalciuria is a hallmark of Dent’s disease and is the major risk factor for stone formation and nephrocalcinosis, as these patients excrete normal quantities of oxalate, citrate, uric acid, and other stone-risk determinants [39, 40]. Infants and young children a have higher degree of calcium excretion, in the range of 10–12 mg/kg, compared with adults, with 4–6 mg/kg, and the hypercalciuria persist while fasting [31]. The hypercalciuria in Dent’s disease is responsive to dietary calcium restriction and thiazide diuretics [42].

##### Hereditary hypophosphatemic rickets with hypercalciuria (HHRH)

Tieder et al. [43] reported a Bedouin tribe with hypercalciuria, hypophosphatemic rickets, and elevated calcitriol levels. Patients with this disease have decreased tubular absorption of phosphate and consequently hypophosphatemia, high serum levels of 1,25-(OH)_2_D_3_, and enhanced intestinal absorption of calcium, resulting in hypercalciuric nephrocalcinosis and urolithiasis. Three types of sodium-phosphate cotransporter have been found in the renal proximal tubule [type I, type IIa, and type III sodium/phosphate (Na/Pi) cotransporters] [44]. Type I Na/Pi cotransporter is present on the brush border membrane and has distinct anion channel properties. It is not regulated under normal physiological conditions and is not believed to be a major determinant of phosphorous reabsorbtion by the proximal tubule. The type IIa Na/Pi cotransporter (NPT2a) is highly expressed in the brush border membrane and has been shown to influence 70–80% of the Na/Pi cotransporter activity at the brush border membrane [45]. NPT2a is believed to be the major determinant of phosphorous reabsorbtion by the proximal tubule and thus of the tubular reabsorption for phosphate per liter glomerular filtration rate (TP/GFR) value [45, 46]. Absence of renal NPT2a expression leads to increased renal phosphate loss resulting in hypophosphatemia [45]. The NPT2a is inhibited by PTH and FGF-23 [47–49]. The type IIb Na/Pi cotransporter is expressed in the small intestine and other epithelial cells but not in the kidney. The type IIc Na/Pi cotransporter is expressed in the brush border membrane of the proximal tubule in weaning animals and decreases with age [50, 51]. The type III Na/Pi cotransporter (Glvr-1 and Ram-1) is expressed at the basolateral aspect of the proximal tubule and is believed to play a housekeeping role in maintaining cellular Pi concentration. Tenenhouse et al. [52] showed that Npt1, Npt2, Glvr-1, and Ram-1 account for approximately 15%, 84%, 0.5%, and 0.5%, respectively, of total Na^+^/Pi cotransporter mRNA in the mouse kidney. Prie et al. [53] sequenced the NPT2a gene from 20 patients with urolithiasis or bone demineralization associated with idiopathic hypophosphatemia and found two patients with NPT2a mutations, one with a substitution of phenylalanine for arginine 48 (exon 3) and the second with a methionine for valine 147 substitution (exon 5); both patients were heterozygous for these mutations. Lapointe et al. [54] found no disease-causing mutation in NPT2a in a cohort of recurrent hypercalciuric stone formers with a TP/GFR of ≤0.7 mmol/l. Although NPT2a appeared to be a good candidate gene for HHRH, Jones et al. [55] and van den Huevel et al. [56] found no mutation in the gene for NPT2a. Only recently have SLC34A3 mutations in Na^+^/Pi-IIc cotransporter been described in patients with HHRH [57].

#### Thick ascending loop of Henle in hypercalciuria

The permeability of calcium is very low in the thin descending and ascending loop of Henle. In TALH, where ∼20% of calcium is absorbed, calcium absorption is passive and driven by the large lumen-positive potential created by sodium absorption [26, 27]. Paracellin-1 in the TALH plays a critical role in control of passive calcium absorption. We discuss briefly the recent developments in Bartter syndrome (and calcium-sensing receptor), and familial hypomagnesemia with hypercalciuria and nephrocalcinosis (FHHNC), which have improved the understanding of calcium homeostasis in this segment of the nephron (Fig. 2).

##### Bartter syndrome

Bartter syndrome is a rare, genetically heterogeneous, renal tubular disorder secondary to defects in the transepithelial sodium chloride transport across TALH. This autosomal recessive syndrome is characterized phenotypically by failure to thrive, hypokalemia, metabolic alkalosis, secondary hyperaldosteronism with normal blood pressure, increased urinary prostaglandins excretion, and hypercalciuria with nephrocalcinosis [58]. A breakdown in sodium absorption in Bartter syndrome leads to poor paracellular absorption of calcium, leading to hypercalciuria. Bartter syndrome can occur from mutation in one of the five genes: (a) Bartter type 1 from mutations in the gene encoding for the luminal bumetanide-sensitive NKCC2 (gene *SLC12A1*, locus 15q15), (b) Bartter type 2 from mutations in the gene encoding for the luminal potassium channel ROMK(*KCNJ1*, locus 11q24), (c) Bartter type 3 from mutations in the gene encoding for the voltage-gated chloride channel on the basolateral membrane (CLC-Kb; gene *CLCNKB*, locus 1p36), (d) Bartter type 4 from mutations in the gene encoding for Barttin, a beta subunit required for trafficking of CLC-Kb and CLC-Ka on the basolateral membrane (Barttin; *BSND* gene, locus 1p31), and (e) Bartter type 5 from activating mutations in the gene encoding for the calcium-sensing receptor located on the basolateral membrane (CaSR; *CASR* gene, locus 3q13) [59–64].

The clinical presentation of Bartter syndrome can be “classical”, or more severe presenting in the perinatal period. Because of the defect in sodium handling by the TALH, there is a failure in the paracellular absorption of calcium, causing hypercalciuria and kidney-stone formation with or without nephrocalcinosis. Children with type 3 Bartter syndrome can exhibit a mixed Bartter-Gitelman phenotype consistent with the role of this chloride ion channel in both the TALH and DCT. Type 4 Bartter syndrome is associated with sensorineural deafness given the role of Barttin, CLC-Ka, and CLC-Kb in the marginal cells of the scala media of the inner ear [62].

##### Calcium-sensing receptor (CaSR)

The CASR gene, located on chromosome 3q13.3-q21, encodes for a plasma membrane G-protein-coupled receptor known as the calcium-sensing receptor (CaSR), which is stimulated by divalent ions such as calcium and magnesium [65]. The CaSR plays a role in regulation of PTH secretion and in renal tubular calcium reabsorption in response to alterations in extracellular calcium concentrations. It is expressed in the basolateral side of the cortical TALH, and its stimulation by elevated peritubular levels of reabsorbed calcium induces an inhibition of NKCC2 and ROMK, resulting in decreased sodium absorption and subsequently calcium absorption [65–67]. Pearce et al. [68] described six kindreds with an autosomal dominant inheritance of hypocalcemia and hypercalciuria resulting from an activating mutations of the CASR gene. Activating mutations of the CASR gene give rise to hypercalciuria and hypocalcemia because of the direct effect in TALH cells (where the CaSR can inhibit calcium absorption) and to the inhibition of PTH secretion, which induces additional downregulation of calcium absorption in the distal tubule. The inhibition of the NKCC2 transporter and ROMK channel leads to a Bartter syndrome type 5. Children will present with hypocalcemia (usually mild and asymptomatic but at times with carpopedal spasm and seizures), hypercalciuria, and polyuria, and about half may have associated hypomagnesemia [68–70]. The key feature to note is that therapy of hypocalcemia with vitamin D or calcium dramatically increases urinary calcium excretion. This will further lead to polyuria, nephrocalcinosis, nephrolithiasis, and reduction in renal function, which may be partially reversible following cessation of treatment [68]. Thus, it is important to identify subjects with gain-of-function CASR mutation, and in these patients, Vitamin D therapy should be restricted to symptomatic patients only, with careful follow-up of urine calcium excretion and consideration of anticalciuric diuretics [71].

##### Familial hypomagnesemia with hypercalciuria and nephrocalcinosis (FHHNC)

FHHNC, or Michelis-Castrillo syndrome [72], is a rare tubular disorder. It is inherited as an autosomal recessive disorder causing mutations in the *PCLN-1* gene on 3q27, which encodes for the protein claudin 16/paracellin-1 [73, 74]. Claudins are membrane proteins that are believed to play an important role in the integrity of the tight junction. Paracellin-1, a member of the claudin family, is expressed in the tight junctions of the TALH in humans [75]. The defect in paracellin-1 function interferes with the paracellular absorption of calcium and magnesium in the TALH.

FHHNC presents at birth. It is characterized by magnesium and calcium wasting, resulting in persistent hypomagnesemia (presents with neonatal seizures), marked hypercalciuria leading to early nephrocalcinosis, incomplete distal renal tubular acidocis (dRTA), hypocitraturia, urinary tract infections, polyuria, and progressive renal failure [73–77]. Some children with FHHNC have ocular abnormalities, such as severe myopia, nystagmus, and chorioretinitis [78]. Children reach end stage by their teenage or young-adult years. The serum calcium level remains in the normal range. Hypocalcemia is possibly prevented by increased transcellular tubular calcium absorption in the distal tubule, intestinal calcium absorption, and calcium release out of bone, mediated by different hormones such as 1,25-(OH)_2_-D_3_ and PTH. The serum PTH is elevated during the course of disease and precedes the impairment in GFR. The reduced concentrating ability, incomplete dRTA, recurrent urinary tract infections, and the development of renal insufficiency are not believed to result directly from the genetic defect but rather as a consequence of medullary interstitial damage from nephrocalcinosis. Children with FHHNC do not have clinically significant salt wasting or hypokalemic metabolic alkalosis and have normal renin and aldosterone levels, which helps to differentiate this entity from Bartter syndrome [75, 79]. The elevated serum PTH helps in differentiating it from Dent’s disease.

#### Distal renal tubule and hypercalciuria

The fine tuning of calcium excretion occurs in the distal part of the nephron in the DCT, connecting tubule and cortical collecting duct. Calcium absorption occurs in the principal cells through an active transcellular pathway (Fig. 3). Pseudohypoaldosteronism (PHA) type II from with-no-lysine kinase 4 (WNK-4) mutation can cause hypercalciuria from its role in regulation of TRPV5, whereas dRTA leads to hypercalciuria indirectly from metabolic acidosis and increased bone resorption.

##### Pseudohypoaldosteronism type II (PHA II)

PHA type II is a genetic disorder due to mutations in the gene encoding WNK-1 or -4, which produces a clinical phenotype of hypertension, hyperkalemia, and metabolic acidosis. Mayan et al. [80] described a family with WNK4 gene mutation was associated with marked hypercalciuria (and osteopenia) that was responsive to thiazide diuretics. Jiang et al. [81] recently showed that WNK4 positively regulates TRPV5-mediated calcium transport, which could account for the observed hypercalciuria.

##### Primary distal renal tubular acidosis (dRTA)

Primary dRTA is a hereditary disorder characterized by impaired renal acid secretion resulting in hyperchloremic metabolic acidosis, hypokalemia, hypercalciuria, hypocitraturia, and inappropriately high urinary pH. The acidification of urine at the distal tubule involves multiple proteins composed of the vacuolar H^+^-ATPase, the band 3-anion exchanger 1 (AE1) (${{\text{Cl}}^{ - } } \mathord{\left/ {\vphantom {{{\text{Cl}}^{ - } } {{\text{HCO}}^{ - }_{3} }}} \right. \kern-\nulldelimiterspace} {{\text{HCO}}^{ - }_{3} }$), and carbonic anhydrase II. Thus, dRTA can occur following mutation in the SLC4A1gene for the AE1 in autosomal dominant dRTA [82, 83], in the gene *ATP6V1B1* coding for β subunit of the vacuolar H^+^-ATPase (located in chromosome 2p13) in autosomal recessive dRTA with sensorineural deafness [84, 85], and in the gene *ATP6VoA4* (located on chromosome 7q33-34) for α subunit of the vacuolar H^+^-ATPase in autosomal recessive dRTA without sensorineural deafness [86]. Mixed RTA can arise from mutation in *CAII* gene for carbonic anhydrase II enzyme [87, 88]. Primary dRTA produces a profound metabolic acidosis, growth retardation, and impressive hypercalciuria, nephrolithiasis, and nephrocalcinosis. The hypercalciuria is believed to be secondary from the increased buffering function of the bone and direct effect of metabolic acidosis on calcium absorption, and the development of progressive nephrocalcinosis is further aggravated by the associated hypocitraturia.

## Idiopathic hypercalciuria: absorptive, renal, and resorptive hypercalciuria (role of vitamin D, vitamin D receptor, PTH, and cytokines)

Pak et al. [89] introduced a tripartite classification of absorptive, renal, and resorptive hypercalciuria for idiopathic hypercalciuria. Over the years, investigators have further modified the classification based on urine calcium excretion, serum phosphate, and serum PTH secretion during fasting and after a calcium load [90]. It is now postulated that idiopathic hypercalciuria can occur from either or in combination with an (1) increased intestinal calcium absorption mediated either by a direct increase in calcium absorption (type 1 absorptive hypercalciuria) or through excess 1,25-(OH)_2_D_3_-mediated calcium absorption (type II absorptive hypercalciuria); (2) decreased renal absorption of either calcium (renal hypercalciuria) or phosphorus (type III absorptive hypercalciuria); (3) enhanced bone resorption (resorptive hypercalciuria) [91–93]. Maierhofer et al. [94] showed that administration of 1,25-(OH)_2_D_3_ while eating a normal-calcium diet in healthy subjects led to an increase in intestinal calcium absorption and an increase in urinary calcium excretion, concluding that the key components of idiopathic hypercalciuria are related to calcitriol. They also showed that increased calcitriol administration in humans on a calcium restricted diet leads to negative calcium balance from increased urinary calcium loss mediated through increased bone resorption [95]. Similarly, vitamin D toxicity gives rise to hypercalcemia and hypercalciuria by stimulating intestinal calcium absorption. It is important to remember that hypercalciuria usually precedes hypercalcemia as an indicator of vitamin D overdose [96]. A fair number of investigators have observed that blood calcitriol concentration is, on average, higher in patients with idiopathic hypercalciuria or inappropriately normal for the condition compared with healthy subjects. Kaplan et al. [97] found elevated calcitriol levels in one third of absorptive hypercalciuria patients and normal values in two thirds, which may be considered as inappropriately high given the presence of relative hypoparathyroidism. Zerwekh and Pak [98] observed that in both renal and absorptive hypercalciuric subjects on thiazides, urine calcium excretion was normalized, but only the renal hypercalciuric group showed a decrease in intestinal hyperabsorption, PTH, and calcitriol level, whereas no changes were observed in the absorptive hypercalciuric subjects. These results would support different inciting events in development of hypercalciuria, namely, primary calcium leak in renal hypercalciuria and abnormal 1,25-(OH)_2_-D_3_ metabolism in absorptive hypercalciuria.

Data from NPT2a ^−^/^−^ mice that lack the Na^+^/Pi cotransporter have provided further insight into the role of 1,25-(OH)_2_-D_3_ in the development of hypercalciuria. The primary defect in tubular phosphate absorption in NPT2a ^−^/^−^ mice stimulates calcitriol synthesis by the kidney, which in turn increases intestinal absorption of calcium and phosphate and inhibits PTH secretion, resulting in hypercalciuria [45]. A disruption in the 1 α-hydroxylase gene in NPT 2a^−^/^−^ mice decreases urinary calcium excretion and prevents development of nephrolithiasis, signifying the importance of increased calcitriol synthesis in the development of hypercalciuria [99]. Similarly, in subjects with hypophosphatemic tubular disorder such as X-linked hypophosphatemic rickets (PHEX) and autosomal dominant hypophosphatemic rickets (FGF-23) with decreased production of calcitriol due to the inhibitory effect of FGF-23, no hypercalciuria is observed until therapy with calcitriol is instituted [100, 101]. Studies on the genetic hypercalciuric stone-forming (GHS) rat model suggest a role for an increase in number and/or function of vitamin D receptors (VDR) in enterocyte [102, 103]. Favus et al. [104] found an elevation in tissue VDR level in patients with idiopathic hypercalciuria with normal calcitriol level.

**Fig. 4:**
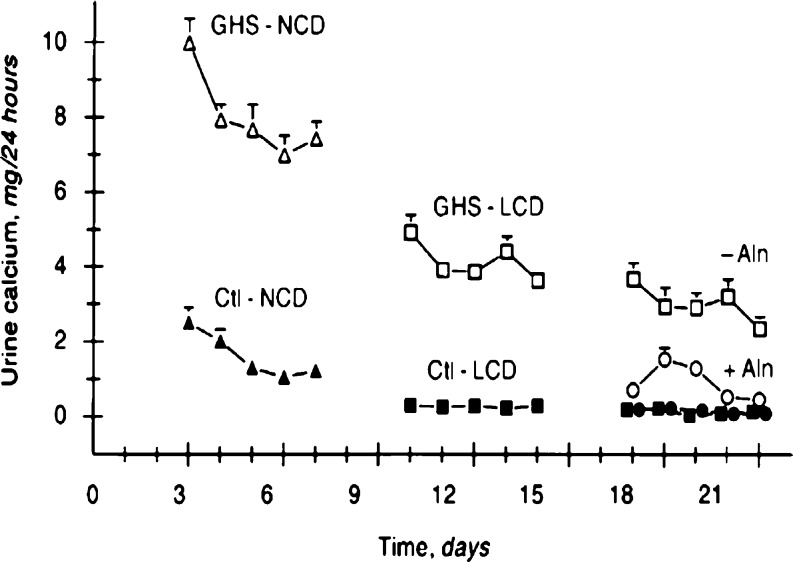
Urine calcium excretion in genetic hypercalciuric stone-forming (GHS) and control (Ctl) rats. Alendronate led to a significant decrease in urine calcium in the GHS, but not in the Ctl rats. GHS rats (*open symbols*), Ctl rats (*closed symbols*), *NCD* normal-calcium diet (1.2% calcium, *triangles*), *LCD* low-calcium diet (0.02% calcium, *squares*), *LCD + Aln* alendronate (Aln, 50 g/kg per 24 h, *circles*). Used with permission [106]

From studies done on GHS rats, it seems that the role of bone in the development of hypercalciuria appears to be as equally important as the intestines. While on a low-calcium diet, GHS rats continue to have markedly elevated urine calcium excretion exceeding their dietary intake, suggesting a role for increased bone resorption. Krieger et al. [105] showed that bone in GHS rats is sensitive to 1,25-(OH)_2_D_3_-induced bone resorption, and Bushinsky et al. [106] showed that alendronate decreases urine calcium excretion in GHS rats on a low-calcium diet to a level below their dietary intake (Fig. 4). Weisinger et al. [107] found alendronate to decrease urine calcium excretion in adults with hypercalciuria, and Freundlich and Alon [abstract to be presented at 14th International Pediatric Nephrology Association (IPNA) 2007 meeting] recently reported preliminary similar outcome in osteopenic hypercalciuric children. Cytokines are known to induce bone resorption and inhibit bone formation and may play a role in the rare of “resorptive hypercalciuria”, in which a primary bone disorder is believed to be the inciting defect. Ghazali et al. [108] found cytokines such as interleukin (IL)-1β, IL-6, tumor necrosis factor (TNF)-α, and granulocyte, macrophage stimulating factor to be increased in hypercalciuric calcium-stone-forming subjects with increased bone loss. Similar findings have been reported by Pacifici et al. [109], which allude to a role for cytokines in the development of hypercalciuria.

Hypercalciuria is a complex *polygenic* trait, and it is possible that absorptive and renal forms of hypercalciuria may represent a continuum of a single disease [110, 111]. When adults with idiopathic hypercalciuria are placed on a low-calcium diet, there is a continuum from those who are in “positive calcium balance”, suggesting a component of direct increased intestinal calcium absorption, to those in “negative calcium balance”, suggesting other mechanisms of hypercalciuria [110]. The lack of evidence of increased bone turnover in children with hypercalciuria suggests that renal and absorptive hypercalciuria may not be distinct physiologic entities [112]. An oral calcium loading test was popular in the past to differentiate between the different forms of idiopathic hypercalciuria; it has recently come under challenge [91, 113]. In children, Aladjem et al. [114] reevaluated calcium-loading tests after an interval of 3–7 years in children who were initially diagnosed as having absorptive or renal hypercalciuria and found a different result in more than half of the children studied. However, the classification, although not practical, has allowed investigators to develop a structured approach to the understanding of hypercalciuria.

## Association of hypercalciuria with osteoporosis

A cross-sectional study from the Third National Health and Nutrition Examination Survey (NHANES III) showed that men (data weaker for women) with kidney-stone history had lower femoral neck BMD than did men without kidney stones after adjusting for age, body mass index (BMI), ethnicity, and other potential confounders, with a concomitant higher prevalence of wrist and spine fractures [115]. In a prospective study, Asplin et al. [116] found that the severity of urine calcium excretion best predicted bone loss in idiopathic hypercalciuric stone formers. Vezzoli et al. [117] observed lower BMD in hypercalciuric compared with normocalciuric stone-forming women, even in the presence of increased intestinal calcium absorption documented by strontium absorption. Similarly, Pietschmann et al. [118] found lower spinal BMD in hypercalciuric compared with normocalciuric stone formers. In contrast, Jaeger et al. [119] and Barkin et al. [120] found no difference in BMD between normocalciuric and hypercalciuric stone formers.

When hypercalciuric stone formers were studied based on Pak’s classification, the decrease in BMD was more frequent and greater in patients with renal hypercalciuria than in those with absorptive hypercalciuria [120, 121]. Other studies observed no reduction in BMD in absorptive hypercalciuria [122, 123]. The overall trend in the literature on adults with hypercalciuria, with or without stone disease, would suggest that they have lower BMD, but the results may vary based on subgroup analysis such as normocalciuric versus hypercalciuric stone formers or between renal versus absorptive hypercalciuria.

Penido et al. [124] found lower BMD in children with idiopathic hypercalciuria, and in a subsequent study [125], they found that these findings were more marked in children with hypocitraturia in addition to the hypercalciuria. Garcia-Neto et al. [126] had also made similar observation in an earlier study but made an interesting observation of negative linear correlation between age and bone mineral content in children with idiopathic hypercalciuria, namely, the Z score for BMD was much lower in older children with hypercalciuria, which raises the issue of whether adult osteoporosis has its origin in childhood. Similarly, Freundlich et al. [127] showed that reduced BMD was present in children with hypercalciuria and concomitantly found a high incidence of both hypercalciuria and reduced BMD in their asymptomatic mothers. The data in both children and adults indicate that the risk for bone loss is present in patients with hypercalciuria, and its origin may lie in childhood; hence, one must consider monitoring bone density as a proxy for calcium balance in children.

## Diet in hypercalciuria (or physiological hypercalciuria)

Diet can have a significant impact on calcium handling by the renal tubules. Urinary calcium excretion is significantly affected by sodium, protein, potassium, phosphorous, and calcium in the diet. Ninety percent of calcium absorption occurs as a paracellular event in the proximal tubule and TALH, which places calcium absorption at the mercy of sodium absorption. An increase in calcium delivery to the distal nephron for transcellular absorption can overwhelm the distal nephron, leading to obligatory hypercalciuria. An increase in either oral or intravenous sodium chloride inhibits net renal tubular calcium absorption and is used with beneficial effect in the treatment of hypercalcemia to increase urinary calcium excretion. The average consumption of salt in industrialized countries is 10 g (or 170 mmol Na)/day per person as determined by urinary 24-h excretion in the INTERSALT study [128]. Nordin et al. [129] have shown that approximately 1 mmol (or 40 mg) calcium is excreted for every 100 mmol (or 2.3 g) of sodium. There is a reproducible linear positive correlation between urinary sodium (a surrogate for dietary intake) and calcium excretion in stone formers as well as in normal individuals [130]. Thus, a diet high in sodium can lead to hypercalciuria [131].

An increase in dietary protein intake increases net acid excretion because of the release of protons from oxidation of sulfur in the amino acids methionine, cysteine, and cystine [132]. Conversely, dietary potassium found mostly in the form of potassium salts of metabolizable organic anions in vegetable and fruits, reflects the dietary intake of actual bicarbonate or potential bicarbonate, which reduce net acid excretion [133]. Urine calcium excretion increases as net acid excretion increases; hence, it rises progressively as the protein intake increases. The increment in urinary calcium excretion is ∼0.04 mmol (∼1.6 mg) calcium per gram of protein. The increase in calcium excretion with dietary protein is more marked in calcium-stone formers than in healthy subjects [91, 134]. Similarly in healthy subjects, an increase in dietary calcium increases urine calcium excretion by 6–7% of the dietary intake increment, whereas the change in calcium-stone formers is almost twice for the same increase in calcium intake [133]. A severe dietary phosphate deprivation induces increased calcium excretion, probably by activating the vitamin D endocrine system and thereby enhancing intestinal calcium absorption when calcium is available in the diet, or bone resorption when dietary calcium is low [94, 95, 135].

In the management of hypercalciuric stone formers, close attention must be paid to dietary intake and corrections made for dietary errors. Dietary hypercalciuria linked to excessive intakes of sodium, protein, or calcium or to deficiency in potassium or phosphate intake is diagnosed when calcium excretion is high while the patient is on his/her usual diet and normalizes during optimal dietary conditions. Thus, it appears that the more society deviates from the traditional balanced diet with optimal intake of protein, salt, fruits, and vegetables, replacing them with sodium-rich fast foods and artificial drinks, accompanied by a decrease in intake of potassium-rich fruits and vegetables, and increase in protein intake, the higher is the risk for “physiological hypercalciuria” [136].

## Approach to and management of hypercalciuria

Hypercalciuria in children can present as nonglomerular hematuria (gross or microscopic), noninfectious dysuria, urinary frequency and dysuria, abdominal and back pain, or with urolithiasis [137, 138]. It can be intermittent or persistent, a transient phenomenon or associated with a family history of urolithiasis. Once hypercalciuria is detected in a child, a secondary etiology should be considered, as successful correction of hypercalciuria in such cases depends on eradication of the primary cause. An evaluation for the rare monogenic disorders characterized by hypercalciuria should be considered in the presence of positive family history, failure to thrive, growth retardation, rickets, acid-base disturbances, renal dysfunction, proteinuria, electrolyte imbalance, dysmorphic features, or poor response to therapy.

Hypercalciuria, like blood pressure, is defined in children as urinary calcium excretion of >4 mg/kg per day on a “statistical” basis, whereas in adults, hypercalciuria is defined as >250 mg/day in women and >300 mg/day in men as an “outcome” value observed in most calcium-stone formers. The statistical cutoffs of 24-h urine >4 mg/kg per day or urine calcium/creatinine ratio >0.21 and their clinical implications have been recently addressed in detail by Butani and Kalia [139]. They raise many questions about planning a strategy for therapy in children. We believe that only symptomatic hypercalciuric children should be treated with pharmacological agents, whereas nonpharmacological intervention (vide infra) can be used more liberally.

When idiopathic hypercalciuria is confirmed in symptomatic children, we recommend as the first step to assess whether dietary manipulation can normalize calcium excretion. We recommend a Dietary Reference Intake for protein and calcium that is not excessive in salt (2.0–2.4 g) per day and supplemented with at least the recommended daily allowance of five to six servings of fruits and vegetables (3.0–3.5 g potassium) per day. Compliance with these dietary recommendations can be assessed by measuring urine Na^+^/K ratio, which should be <2.5. The dietary implications of salt, protein, and fruits and vegetables in hypercalciuria are well known, but we are cognizant of the fact that children may not fully comply with such dietary manipulations nor with the traditional recommendation of high fluid intake [4]. If in 4–6 weeks hypercalciuria persists, treatment with potassium citrate at 1–1.5 mEq of potassium per kilogram per day is recommended. If the child fails to tolerate potassium citrate or hypercalciuria fails to correct, a thiazide diuretic can be added [4, 136]. Chlorothiazide 15–25 mg/kg per day or hydrochlorothiazide 1.5–2.5 mg/kg per day can be used. In the past, it was proposed that thiazide-induced hypocalciuria occurred from volume contraction, which through increased proximal sodium absorption would increase the passive calcium absorption. Costanzo et al. [140] showed that acute administration of chlorothiazide in the tubular lumen stimulated transcellular calcium transport in the DCT. The earlier observation made by Costanzo et al. was recently confirmed by Jang et al. [141], who showed that thiazides increased the expression of TRPV5 and calbindin-D(28K) and decreased expression of sodium-chloride cotransporter in the DCT, leading to increased calcium absorption in the DCT. Children on long-term thiazide diuretics will need to be monitored for dyselectrolytemia, hyperlipidemia, and hyperglycemia. One can consider adding amiloride, as it further increases the hypocalciuric effect and decreases potassium and magnesium loss. Contrary to past practice, dietary restriction of calcium is not recommended in children with hypercalciuria, as it puts the growing child at risk for negative calcium balance and poor bone mineralization. It may also increase urinary excretion of oxalate from increased gastrointestinal absorption of oxalate resulting from decreased luminal calcium present to bind with oxalate. For risk of possible negative calcium balance, drugs such as sodium cellulose phosphate, a nonabsorbable ion-exchange resin used for complexing intestinal calcium, are not used in children. Phosphate salts can be used in children with hypercalciuria due to tubular phosphate leak. In children with hypercalciuria secondary to renal tubular acidosis, potassium citrate is the drug of choice for treatment of hypercalciuria. At times, this may need to be supplemented by sodium bicarbonate and calcium-sparing diuretics.

In summary, a better understanding of the rare inherited renal tubular disorders associated with hypercalciuria has improved our understanding of calcium handling by the kidney and development of hypercalciuria. On the other hand, our understanding of the more common idiopathic hypercalciuria, probably inherited as a polygenic trait and affected by the environment, remains dismal, and even more so in children. Many questions remain open: Is dietary manipulation adequate for all children, or should a subset of children be offered anticalciuric therapy given that dietary manipulation will not suffice or is not needed in them? Should all children be treated with anticalciuric therapy? Once an anticalciuric therapy is initiated, then for how long should it be continued? The data on BMD is suggestive of poor bone health in hypercalciuria; therefore, should all children have a dual-energy X-ray absorptiometry (DXA) scan with all its known pitfalls in children? Should DXA findings be considered in planning therapy for hypercalciuria in children? There are many such questions with respect to hypercalcuria in children that need to be addressed. We encourage the pediatric nephrology community to further address these issues in the hope of developing evidence-based care.
